# Heat Transfer of Hybrid Nanomaterials Base Maxwell Micropolar Fluid Flow over an Exponentially Stretching Surface

**DOI:** 10.3390/nano12071207

**Published:** 2022-04-04

**Authors:** Piyu Li, Faisal Z. Duraihem, Aziz Ullah Awan, A. Al-Zubaidi, Nadeem Abbas, Daud Ahmad

**Affiliations:** 1 School of Mathematics and Statistics, Xuzhou University of Technology, Xuzhou 221018, China; pylixzit@126.com; 2 Department of Mathematics, College of Science, King Saud University, Riyadh 11451, Saudi Arabia; faldureiham@ksu.edu.sa; 3 Department of Mathematics, University of the Punjab, Lahore 54590, Pakistan; daud.math@pu.edu.pk; 4 Department of Mathematics, College of Science, King Khalid University, Abha 61413, Saudi Arabia; a.abdoya@kku.edu.sa; 5 Department of Mathematics, Quaid-I-Azam University Islamabad 44000, Pakistan; nabbas@math.qau.edu.pk

**Keywords:** boundary layer flow, micropolar hybrid nanofluid, exponential stretching surface, numerical technique

## Abstract

A numerical investigation of three-dimensional hybrid nanomaterial micropolar fluid flow across an exponentially stretched sheet is performed. Recognized similarity transformations are adopted to convert governing equations from PDEs into the set ODEs. The dimensionless system is settled by the operating numerical approach bvp4c. The impacts of the nanoparticle volume fraction, dimensionless viscosity ratio, stretching ratio parameter, and dimensionless constant on fluid velocity, micropolar angular velocity, fluid temperature, and skin friction coefficient in both *x*-direction and *y*-direction are inspected. Graphical outcomes are shown to predict the features of the concerned parameters into the current problem. These results are vital in the future in the branches of technology and industry. The micropolar function $\;R\left(\eta \right)$ increases for higher values of the micropolar parameter and nanoparticle concentration. Micropolar function $R\left(\eta \right)$ declines for higher values of the micropolar parameter and nanoparticle concentration. Temperature function is enhanced for higher values of solid nanoparticle concentration. Temperature function declines for higher values of the micropolar parameter. The range of the physical parameters are presented as: $0.005<\phi_{2}<0.09,\;Pr=6.2,\;0<K<2,\;0<a<2.0,\;\phi_{1}=0.1,\;\mathrm{and}\;0<c<1.5$.

## 1. Introduction

The micropolar theory was inspected as a theoretical model, but currently, it is animated with several applications. Micropolar fluids, in particular, have found a niche in the field of modeling liquid crystals with magnetic fluids, stiff molecules, muddy liquids, and biological fluids [1]. The classical Navier–Stokes model is utilized to analyze the micropolar fluid, and the microrotation vector is extensively used to define microphenomena. In mathematics, the micropolar fluid model is studied in two opposite directions: one examines incompressible flow, and the other investigates compressible flows. Micropolar sap has been extensively studied for incompressible flow [2], but there are still several issues. The micropolar fluid for compressible flow has been deliberated in a few years (see [3,4,5,6]).

Hybrid nanofluids are a novel type of nanofluid that contains a finite range of metallic nanoparticles and nonmetallic nanoparticles. Using hydrogen reduction technique, Jeena et al. [7] organized a composite of alumina–copper from CuO and Al_2_O_3_ mixture. Suresh et al. [8] described a symbolic expansion in viscosity which increase in thermal conductivity was lower than the variation in thickness. Senthilraja et al. [9] observed the thermal conductivity of nanomaterial and hybrid nanomaterial experimentally with base fluid. For the hybrid nanofluids, natural convection causes a change in heat transfer as see in Refs. [10,11,12]. Suresh et al. [13] obtained a maximum Nusselt number augmentation of 13.56% for $Cu–Al_{2}O_{3}$ hybrid nanofluid. Hemmat et al. [14] used silver and magnesium with water-based hybrid nanofluid in the presence of the nanoparticle volume fraction, which lies between 0% and 2%. Moghadassi et al. [15] investigated the effects of $Cu–Al_{2}O_{3}$ and $Al_{2}O_{3}$ with base fluid water hybrid nanofluid with 0.1% volume fraction on laminar-driven convective heat transmission. In a porous medium, the natural convection of the hybrid nanofluids was examined by Mehryan et al. [16]. Ismael et al. [17] investigated the viscous dissipation and mixed convection of hybrid nanoparticles in a lid-driven cavity. Nadeem et al. [18] studied the effects of MHD with carbon nanofluid over curved surfaces. Nadeem and Abbas [19] highlighted the effects of a modified nanofluid model under time-dependent properties at porous surfaces. Nadeem et al. [20] discussed hybrid nanofluid over a curved surface. Awan et al. [21] worked at an unsteady oblique stagnation point for nanofluid. Awan et al. [22] highlighted the effects of an MHD unsteady oblique stagnation point for second-grade fluid at an oscillatory stretching surface. The micropolar fluid flow over a Riga surface was analyzed by Nadeem et al. [23]. Many researchers have conducted a lot of work on stretching surfaces; interested readers can see [24,25,26,27,28].

The current discussion talks about an steady, incompressible, three-dimensional boundary layer flow of micropolar hybrid nanofluid passing through an exponentially stretching sheet. Recognized similarity transformations are adopted to convert modeled equations from PDEs into a set of ODEs. The reconstructed equations are then solved by the operating numerical approach BVP4C. The impacts of the nanoparticle volume fraction, dimensionless viscosity ratio, stretching ratio parameter, and dimensionless constant on fluid velocity, micropolar angular speed, temperature gradient, and skin friction index in both x−direction and y−direction have been inspected through tables and graphs.

## 2. Mathematical Formulation

**Figure 1:**
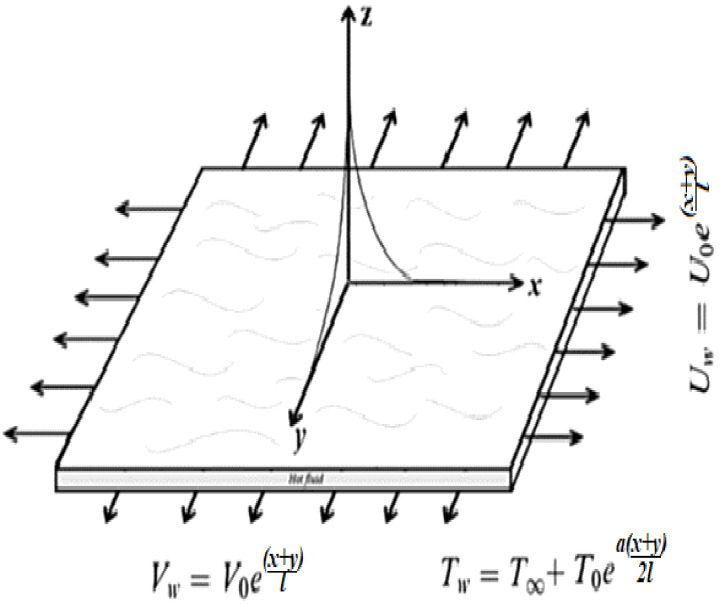
Flow pattern of micropolar hybrid nanofluid.

Here, a steady, incompressible, 3-D boundary layer flow of micropolar hybrid nanomaterial over an exponentially expanding sheet is taken into account, as revealed in Figure 1.

We assumed that the temperature at the wall of the stretching sheet is $T_{w}$, whereas $U_{w}$ and $V_{w}$ are velocities at the wall of the stretching sheet along x-axis and y-axis, respectively. Assumptions of the problem are as follows:

- Three-dimensional flow;
- Micropolar fluid;
- Two-phase model (nanofluid model);
- Exponential stretching sheet;
- Thermal slip.

The mathematical equations for three-dimensional flow are derived using boundary layer assumptions as (see [23,24,25]):

(1) $$\frac{\partial w}{\partial z}+\frac{\partial v}{\partial y}+\frac{\partial u}{\partial x}=0,$$

(2) $$w\frac{\partial u}{\partial z}+v\frac{\partial u}{\partial y}+u\frac{\partial u}{\partial x}=\left(\frac{\kappa +\mu_{hnf}}{\rho_{hnf}}\right)\frac{\partial^{2}u}{\partial z^{2}}+\frac{\kappa }{\rho_{hnf}}\frac{\partial N_{2}}{\partial z},$$

(3) $$w\frac{\partial v}{\partial z}+v\frac{\partial v}{\partial y}+u\frac{\partial v}{\partial x}=\left(\frac{\kappa +\mu_{hnf}}{\rho_{hnf}}\right)\frac{\partial^{2}v}{\partial z^{2}}-\frac{\kappa }{\rho_{hnf}}\frac{\partial N_{1}}{\partial z},$$

(4) $$j\rho_{hnf}\left(+w\frac{\partial N_{1}}{\partial z}+v\frac{\partial N_{1}}{\partial y}+u\frac{\partial N_{1}}{\partial x}\right)=\frac{\partial }{\partial z}\left(\gamma_{hnf}\frac{\partial N_{1}}{\partial z}\right)-\kappa \left(\frac{\partial v}{\partial z}+2N_{1}\right),$$

(5) $$j\rho_{hnf}\left(u\frac{\partial N_{2}}{\partial x}+v\frac{\partial N_{2}}{\partial y}+w\frac{\partial N_{2}}{\partial z}\right)=\frac{\partial }{\partial z}\left(\gamma_{hnf}\frac{\partial N_{2}}{\partial z}\right)-\kappa \left(-\frac{\partial u}{\partial z}+2N_{2}\right),$$

(6) $$w\frac{\partial T}{\partial z}+v\frac{\partial T}{\partial y}+u\frac{\partial T}{\partial x}=\frac{k_{hnf}}{{\left(\rho c_{p}\right)}_{hnf}}\left(\frac{\partial^{2}T}{\partial z^{2}}\right).$$

Associated boundary conditions for three-dimensional flow are:

(7) $$\begin{array}{c} \;w=0,\;v=V_{0}e^{\left(\frac{x+y}{l}\right)},\;u=U_{0}e^{\left(\frac{x+y}{l}\right)},\;N_{1}=\frac{1}{2}\frac{\partial v}{\partial z},\;N_{2}=-\frac{1}{2}\frac{\partial u}{\partial z},\;\\ at\;z\to 0;\;T=T_{w}=T_{\infty }+T_{0}e^{a\left(\frac{x+y}{2l}\right)},\; \end{array}$$

(8) $$\;at\;z\to \infty ;\;u=0,\;v=0,\;N_{1}=0,\;N_{2}=0,\;T=T_{\infty }.$$

Here, u, v, and w are the velocity components along x, y, and z-axes, respectively. $U_{0}$ and $V_{0}$ are the constants, and l is the reference length. $\rho_{hnf}$ and $\mu_{hnf}$ are the density and variable viscosity of hybrid nanomaterial, respectively; κ is the vortex viscosity; $N_{1}$ and $N_{2}$ are the microangular speeds; j is the microinertia, which is defined as $\left(j=\frac{\nu_{f}\;l}{U_{0}e^{\left(\frac{x+y}{l}\right)}}\right)$; $\nu_{f}$ is the coefficient of kinematic viscosity; and $k_{hnf}$ is the hybrid nanofluids’ thermal conductivity, whereas ${\left(C_{p}\right)}_{hnf}$ is the specific heat capacity, and m>0 is a constant that belongs to the interval (0, 1). In the current work, we use *m* = 1/2. The rotational gradient viscosity of a hybrid nanofluid, indicated by $\gamma_{hnf},$ is defined as:

(9) $$\gamma_{hnf}=\left(\mu_{hnf}+\frac{\kappa }{2}\right)j.$$

Some physical properties such as viscosity, density, heat capacity and thermal conductivity for the hybrid nanofluid are expressed in the following Table 1.

## 3. Similarity Variables

Suitable similarity transformations for three-dimensional flow are defined as (see [23,24,26]):

(10) $$\begin{array}{c} \psi \left(x,\;y,\;z\right)=\sqrt{2\nu_{f}lU_{0}}e^{\left(\frac{x+y}{2l}\right)}f\left(\eta \right),\;\phi \left(x,\;y,\;z\right)=-\sqrt{2\nu_{f}lU_{0}}e^{\left(\frac{x+y}{2l}\right)}g\left(\eta \right),\\ \eta =z\sqrt{\frac{U_{0}}{2\nu_{f}l}}e^{\left(\frac{x+y}{2l}\right)},\;u=U_{0}e^{\left(\frac{x+y}{l}\right)}f^{\prime }\left(\eta \right),\;v=V_{0}e^{\left(\frac{x+y}{l}\right)}g^{\prime }\left(\eta \right),\\ w=-\sqrt{\frac{\nu_{f}U_{0}}{2l}}e^{\left(\frac{x+y}{2l}\right)}\left\{f\left(\eta \right)+\eta f^{\prime }\left(\eta \right)+g\left(\eta \right)+\eta g^{\prime }\left(\eta \right)\right\},\\ N_{1}=\sqrt{\frac{U_{0}^{3}}{2\nu_{f}l}}e^{3\left(\frac{x+y}{2l}\right)}R\left(\eta \right),\;N_{2}=\sqrt{\frac{U_{0}^{3}}{2\nu_{f}l}}e^{3\left(\frac{x+y}{2l}\right)}Q\left(\eta \right),\\ \theta =\frac{T-T_{\infty }}{T_{w}-T_{\infty }}\Rightarrow T=T_{\infty }+T_{0}e^{a\left(\frac{x+y}{2l}\right)}\theta \left(\eta \right). \end{array}$$

Making use of suitable transformations, which are defined in Equation (10), our original governing Equations (2)–(6) are transformed into a system of nonlinear ODEs as follows:

(11) $$\left(\frac{\rho_{f}}{\rho_{hnf}}\frac{\mu_{\mathrm{hnf}}}{\mu_{f}}+\frac{\rho_{f}}{\rho_{hnf}}K\right)f^{\prime \prime \prime }+\left(\frac{\rho_{f}}{\rho_{hnf}}K\right)Q^{\prime }-2{\left(f^{\prime }\right)}^{2}-2cf^{\prime }g^{\prime }+\left(1-c\right)\;\eta f^{\prime \prime }g^{\prime }+ff^{\prime \prime }+gf^{\prime \prime }=0,$$

(12) $$\left(\frac{\rho_{f}}{\rho_{hnf}}\frac{\mu_{hnf}}{\mu_{f}}+\frac{\rho_{f}}{\rho_{hnf}}K\right)g^{\prime \prime \prime }-\left(\frac{\rho_{f}}{c\rho_{hnf}}K\right)R^{\prime }-2f^{\prime }g^{\prime }-2c{\left(g^{\prime }\right)}^{2}+\left(1-c\right)\;\eta g^{\prime }g^{\prime \prime }+fg^{\prime \prime }+gg^{\prime \prime }=0,$$

(13) $$\left(\frac{\rho_{f}}{\rho_{hnf}}\frac{\mu_{hnf}}{\mu_{f}}+\frac{\rho_{f}}{\rho_{hnf}}\frac{K}{2}\right)R^{\prime \prime }-\left(\frac{2\rho_{f}}{\rho_{hnf}}K\right)\left(cg^{\prime \prime }+2R\right)-3f^{\prime }R-3cg^{\prime }R+\left(1-c\right)\;\eta g^{\prime }R^{\prime }+fR^{\prime }+gR^{\prime }=0,$$

(14) $$\left(\frac{\rho_{f}}{\rho_{hnf}}\frac{\mu_{hnf}}{\mu_{f}}+\frac{\rho_{f}}{\rho_{hnf}}\frac{K}{2}\right)Q^{\prime \prime }+\left(\frac{2\rho_{f}}{\rho_{hnf}}K\right)\left(f^{\prime \prime }-2Q\right)-3f^{\prime }Q-3cg^{\prime }Q+\left(1-c\right)\;\eta g^{\prime }Q^{\prime }+fQ^{\prime }+gQ^{\prime }=0,$$

(15) $$\left(\frac{{\left(\rho c_{p}\right)}_{f}}{{\left(\rho c_{p}\right)}_{hnf}}\frac{k_{hnf}}{k_{f}}\right)\theta^{\prime \prime }-a\;Pr\left(f^{\prime }+cg^{\prime }\right)\theta +Pr\left(1-c\right)\eta g^{\prime }\theta^{\prime }+Pr\left(f+g\right)\theta^{\prime }=0,$$

Related nondimensional boundary conditions for three-dimensional flow are defined as:

(16) $$\begin{array}{c} f\left(0\right)=0,\;g\left(0\right)=0,\;f^{\prime }\left(0\right)=1,\;g^{\prime }\left(0\right)=1,\\ R\left(0\right)=\frac{1}{2}cg^{\prime \prime }\left(0\right),\;Q\left(0\right)=-\frac{1}{2}f^{\prime \prime }\left(0\right),\\ \theta \left(0\right)=1,\;R\left(\infty \right)=0,\;Q\left(\infty \right)=0,\\ f^{\prime }\left(\infty \right)=0,\;g^{\prime }\left(\infty \right)=0,\;\theta \left(\infty \right)=0 \end{array}$$

where all the derivatives are taken concerning η and denoted by $\left(’\right)$, $\left(a\right)$ is the dimensionless parameter, $\left(K=\frac{\kappa }{\mu_{f}}\right)$ is the micropolar parameter,$\left(c=\frac{V_{0}}{U_{0}}\right)$ is the ratio of the stretching rate along the y-direction to the x-direction, $\left(Pr=\frac{{\left(\mu c_{p}\right)}_{f}}{k_{f}}\right)$ is the Prandtl number, and $\phi_{1}$ and $\phi_{2}$ are two nanoparticles whose values are 0.1 and 0.01, respectively, constant in all scenarios. Now the coefficients of skin friction in x-direction and y-direction are defined as:

(17) $$C_{fx}=\frac{\tau_{wx}}{\rho_{hnf}U_{w}^{2}},\;C_{fy}=\frac{\tau_{wy}}{\rho_{hnf}U_{w}^{2}},$$

where $\left(\tau_{wx}\right)$ and $\left(\tau_{wy}\right)$ are defined as:

(18) $$\begin{array}{c} \tau_{wx}=\kappa {\left(N_{2}\right)}_{z=0}+\left(\mu_{hnf}+\kappa \right){\left(\frac{\partial u}{\partial z}\right)}_{z=0},\\ \tau_{wy}=\kappa {\left(N_{1}\right)}_{z=0}+\left(\mu_{hnf}+\kappa \right){\left(\frac{\partial v}{\partial z}\right)}_{z=0}. \end{array}$$

Making use of nondimensional variables, the physical parameters have the form

(19) $$\begin{array}{c} Re_{x}^{\frac{1}{2}}C_{fx}=\frac{\left(\frac{\rho_{f}}{\rho_{hnf}}\frac{\mu_{hnf}}{\mu_{f}}+\frac{\rho_{f}}{\rho_{hnf}}\frac{K}{2}\right)f^{\prime \prime }\left(0\right)}{\sqrt{2}},\\ Re_{y}^{\frac{1}{2}}C_{fy}=\frac{c\left(\frac{\rho_{f}}{\rho_{hnf}}\frac{\mu_{hnf}}{\mu_{f}}+\frac{\rho_{f}}{\rho_{hnf}}\frac{3K}{2}\right)g^{\prime \prime }\left(0\right)}{\sqrt{2}}, \end{array}$$

where the Reynolds number is $Re=\frac{U_{w}l}{\nu_{f}}$.

## 4. Numerical Procedure

In this analysis, the steady, incompressible, 3-D boundary layer flow of the micropolar hybrid nanomaterial over the exponentially expanding sheet is taken into account. To solve the developing mathematical model and to solve the differential equations by using the bvp4c method after converting differential equations into first-order differential equations, thus the reduced higher-order differential system in the initial value problem. The procedure of the numerical technique is defined below:

(20) $$\begin{array}{c} f\left(\eta \right)=y\left(1\right);\;f’\left(\eta \right)=y\left(2\right);f’’\left(\eta \right)=y\left(3\right);f’’’\left(\eta \right)=yy1;\\ g\left(\eta \right)=y\left(4\right);\;g’\left(\eta \right)=y\left(5\right);g’’\left(\eta \right)=y\left(6\right);g’’’\left(\eta \right)=yy2;\\ R\left(\eta \right)=y\left(7\right);\;R’\left(\eta \right)=y\left(8\right);R’’\left(\eta \right)=yy3;Q\left(\eta \right)=y\left(9\right);\;Q’\left(\eta \right)=y\left(10\right);Q’’\left(\eta \right)=yy4;\theta \left(\eta \right)=y\left(11\right);\;\theta ’\left(\eta \right)=y\left(12\right);\theta ’’\left(\eta \right)=yy5; \end{array}$$

(21) $$yy1=-{\left(\frac{\rho_{f}}{\rho_{hnf}}\frac{\mu_{hnf}}{\mu_{f}}+\frac{\rho_{f}}{\rho_{hnf}}K\right)}^{-1}\left(\left(\frac{\rho_{f}}{\rho_{hnf}}K\right)y\left(10\right)-2y\left(2\right)y\left(2\right)-2cy\left(2\right)y\left(5\right),+\left(1-c\right)\;xy\left(3\right)y\left(5\right)+y\left(1\right)y\left(3\right)+y\left(4\right)y\left(3\right)\right),$$

(22) $$yy2=-{\left(\frac{\rho_{f}}{\rho_{hnf}}\frac{\mu_{hnf}}{\mu_{f}}+\frac{\rho_{f}}{\rho_{hnf}}K\right)}^{-1}\left(-\left(\frac{\rho_{f}}{c\rho_{hnf}}K\right)y\left(8\right)-2y\left(2\right)y\left(5\right),-2cy\left(5\right)y\left(5\right)+\left(1-c\right)\;xy\left(5\right)y\left(6\right)+y\left(1\right)y\left(6\right)+y\left(4\right)y\left(6\right)\right),$$

(23) $$yy3=-{\left(\frac{\rho_{f}}{\rho_{hnf}}\frac{\mu_{hnf}}{\mu_{f}}+\frac{\rho_{f}}{2\rho_{hnf}}K\right)}^{-1}\left(-\left(\frac{2\rho_{f}}{\rho_{hnf}}K\right)\left(cy\left(6\right)+2y\left(7\right)\right)-3y\left(2\right)y\left(7\right),-3cy\left(5\right)y\left(7\right)+\left(1-c\right)\;xy\left(5\right)y\left(8\right)+y\left(1\right)y\left(8\right)+y\left(4\right)y\left(8\right)\right),$$

(24) $$yy4=-{\left(\frac{\rho_{f}}{\rho_{hnf}}\frac{\mu_{hnf}}{\mu_{f}}+\frac{\rho_{f}}{2\rho_{hnf}}K\right)}^{-1}\left(-\left(\frac{2\rho_{f}}{\rho_{hnf}}K\right)\left(cy\left(6\right)+2y\left(9\right)\right)-3y\left(2\right)y\left(9\right)-3cy\left(5\right)y\left(7\right)+\left(1-c\right)\;xy\left(5\right)y\left(10\right)+y\left(1\right)y\left(10\right)+y\left(4\right)y\left(10\right)\right),$$

(25) $$yy5=-{\left(\frac{{\left(\rho c_{p}\right)}_{f}}{{\left(\rho c_{p}\right)}_{hnf}}\frac{k_{hnf}}{k_{f}}\right)}^{-1}\left(-a\;Pr\left(y\left(2\right)+cy\left(5\right)\right)y\left(11\right)+Pr\left(1-c\right)xy\left(5\right)y\left(12\right)+Pr\left(y\left(1\right)+y\left(4\right)\right)y\left(12\right)\right),$$

Related nondimensional boundary conditions for three-dimensional flow are defined as:

(26) $$\begin{array}{c} y0\left(1\right);y0\left(4\right);y0\left(2\right)-1;y0\left(5\right)-1;\;y0\left(7\right)-\frac{1}{2}cy0\left(6\right);y0\left(9\right)+\frac{1}{2}y0\left(3\right);\\ y0\left(11\right)-1;yinf\left(2\right);yinf\left(5\right);yinf\left(7\right);yinf\left(9\right);\;yinf\left(11\right). \end{array}$$

## 5. Graphical Results and Discussion

**Figure 2:**
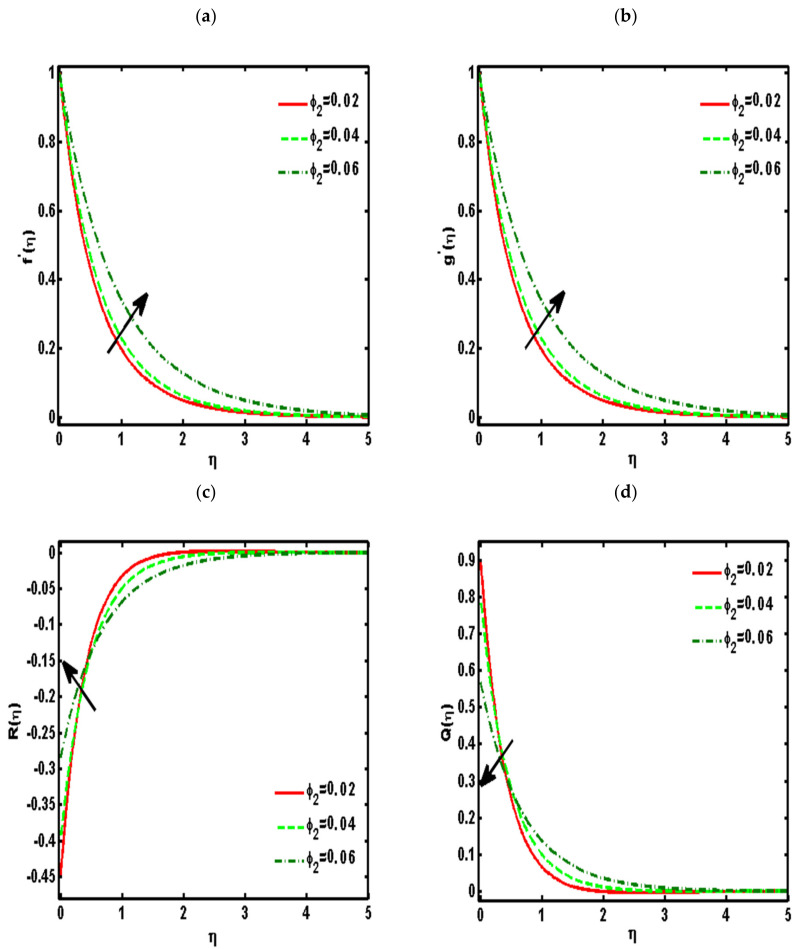
Effects of the nanoparticle volume fraction $\left(\phi_{2}\right)$ on (**a**) $f^{\prime }\left(\eta \right),\;$ (**b**) $g^{\prime }\left(\eta \right),\;$ (**c**) $R\left(\eta \right),\;$ (**d**) $Q\left(\eta \right).$ (Pr=6.2, K=0.5, a=0.5, c=0.5).

**Figure 3:**
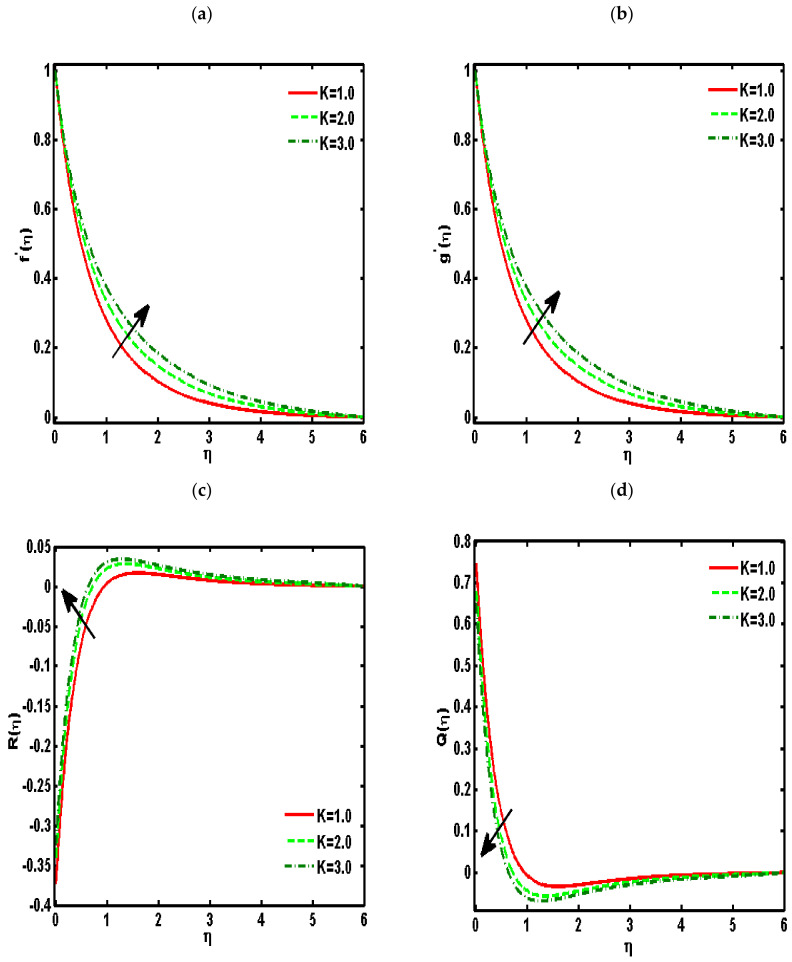
Effects of the dimensionless viscosity ratio $\left(K\right)$ on (**a**) $f^{\prime }\left(\eta \right),$ (**b**) $g^{\prime }\left(\eta \right),$ (**c**) $R\left(\eta \right),$ (**d**) $Q\left(\eta \right)$. ($\phi_{2}=0.01,\;Pr=6.2,\;a=0.5,\;c=0.5$).

**Figure 4:**
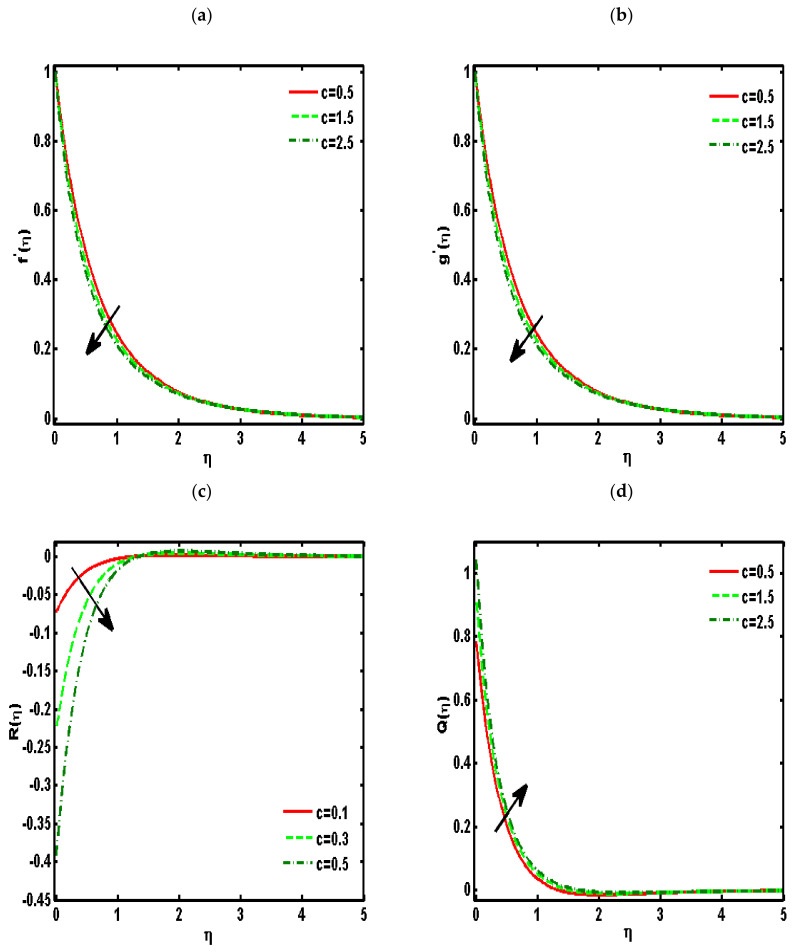
Effects of the stretching ratio parameter $\left(c\right)$ on (**a**) $f^{\prime }\left(\eta \right),\;$ (**b**) $g^{\prime }\left(\eta \right),$ (**c**) $R\left(\eta \right),$ (**d**) $Q\left(\eta \right)$. ($\phi_{2}=0.01,\;Pr=6.2,\;K=0.5,\;a=0.5$).

**Figure 5:**
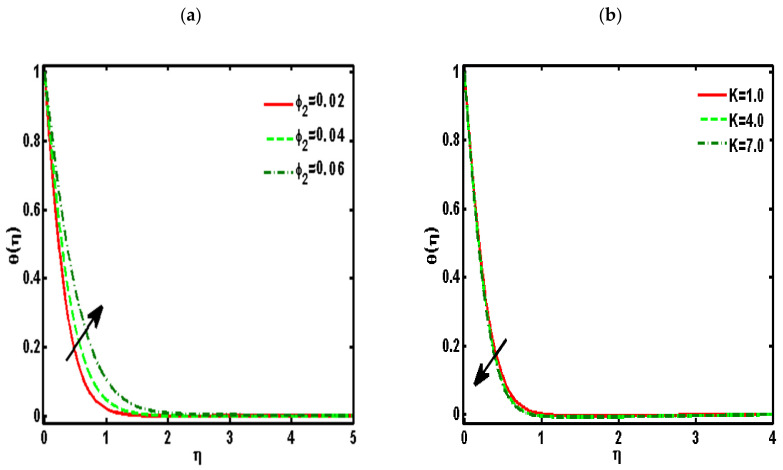
Effects of the (**a**) nanoparticle volume fraction $\left(\phi_{2}\right)$ and (**b**) dimensionless viscosity ratio $\left(K\right)$ on temperature profile $\theta \left(\eta \right)$. (Pr=6.2, a=0.5, c=0.5).

**Figure 6:**
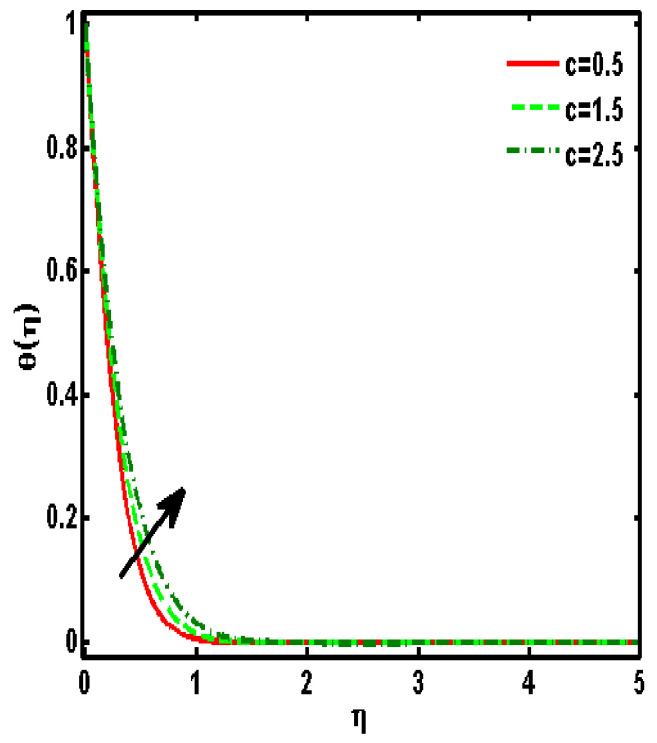
Effects of the stretching ratio parameter $\left(c\right)$ on temperature profile $\theta \left(\eta \right)$. ($\phi_{2}=0.01,\;Pr=6.2,\;K=0.5,\;a=0.5$).

Figure 2, Figure 3, Figure 4, Figure 5 and Figure 6 demonstrated the effects of different parameters, such as the nanoparticle volume fraction $\left(\phi_{2}\right)$, dimensionless viscosity ratio $\left(K\right)$, nondimensional constant $\left(a\right)$, stretching ratio parameter $\left(c\right)$ on $f^{\prime }\left(\eta \right),\;g^{\prime }\left(\eta \right),\;R\left(\eta \right),Q\left(\eta \right),$ and $\theta \left(\eta \right)$. Figure 2a–d presented the effects of solid nanoparticle concentrations on the velocity functions ($f^{\prime }\left(\eta \right)\;\mathrm{and}\;g^{\prime }\left(\eta \right)$) and micropolar functions ($Q\left(\eta \right)\;and\;R\left(\eta \right)$), respectively. It is noted that the velocity function increases for both profiles ($f^{\prime }\left(\eta \right)\;\mathrm{and}\;g^{\prime }\left(\eta \right)$) due to higher values of solid nanoparticle concentrations. The momentum thickness enhances with increasing solid nanoparticles concentrations. The micropolar function $R\left(\eta \right)$ increases for higher values of solid nanoparticle concentrations but declines the micropolar function $Q\left(\eta \right)$ because of higher values of solid nanoparticle concentrations, which are presented in Figure 2c,d. The variation of the micropolar parameter K and velocity functions ($f^{\prime }\left(\eta \right)\;\mathrm{and}\;g^{\prime }\left(\eta \right)$) and micropolar functions ($Q\left(\eta \right)\;and\;R\left(\eta \right)$), respectively, are presented in Figure 3a–d. It is noted that the velocity functions ($f^{\prime }\left(\eta \right)\;\mathrm{and}\;g^{\prime }\left(\eta \right)$) increased due to higher values of the micropolar parameter, which is revealed in Figure 3a,b. As the vertex velocity was enhanced, the movement of the fluid was enhanced. The micropolar function $R\left(\eta \right)$ is enhanced due to increasing values of the micropolar parameter. The micropolar function $Q\left(\eta \right)$ declines for higher values of the micropolar parameter. The variation of the stretching parameter c and velocity functions ($f^{\prime }\left(\eta \right)\;\mathrm{and}\;g^{\prime }\left(\eta \right)$) and micropolar functions ($Q\left(\eta \right)\;and\;R\left(\eta \right)$), respectively, is presented in Figure 4a–d. It is noted that the velocity functions ($f^{\prime }\left(\eta \right)\;\mathrm{and}\;g^{\prime }\left(\eta \right)$) declined due to higher values of the stretching parameter, which is revealed in Figure 4a,b. The micropolar function $R\left(\eta \right)$ declines due to increasing values of the stretching parameter, which is revealed in Figure 4c. The micropolar function $Q\left(\eta \right)$ enhances for higher values of the micropolar parameter, which is revealed in Figure 4d. The impacts of the nanoparticle volume fraction $\left(\phi_{2}\right)$ on temperature profile $\theta \left(\eta \right)$ are demonstrated in Figure 5a. We noticed that for large values of $\left(\phi_{2}\right)$, temperature function $\theta \left(\eta \right)$ increases. Figure 5b shows the influence of $\left(K\right)$ on temperature function $\theta \left(\eta \right)$. It is examined that augmentation in $\left(K\right)$ decreases $\theta \left(\eta \right)$. Figure 6 signifies the influence of the stretching ratio factor $\left(c\right)$ on temperature function $\theta \left(\eta \right)$. It is observed that the nature of the stretching ratio parameter $\left(c\right)$ is the same as the nature of temperature function $\theta \left(\eta \right)$.

In Table 2, the influences of various physical parameters, such as the nanoparticle volume concentration $\left(\phi_{2}\right)$, micropolar parameter $\left(K\right)$, nondimensional constant $\left(a\right)$, and stretching ratio parameter $\left(c\right)$, on the coefficient of skin friction along x-direction and y-direction are illustrated. In Table 2, it is analyzed that for large values of the nanoparticle volume fraction $\left(\phi_{2}\right)$, the skin friction coefficient in both x- and y-directions declines. The effects of the dimensionless viscosity ratio $\left(K\right)$ on $Re_{x}^{\frac{1}{2}}C_{fx}$ and $Re_{y}^{\frac{1}{2}}C_{fy}$ are presented in Table 2. It is realized that increasing (K) decreases the skin friction coefficient in both x- and y- directions. The impacts of the stretching ratio parameter $\left(c\right)$ on $Re_{x}^{\frac{1}{2}}C_{fx}$ and $Re_{y}^{\frac{1}{2}}C_{fy}$ are demonstrated in Table 2. It is recognized that for large values of the stretching ratio parameter $\left(c\right)$, the skin friction coefficient in both directions such that $Re_{x}^{\frac{1}{2}}C_{fx}$ and $Re_{y}^{\frac{1}{2}}C_{fy}$ shows a decaying nature. The effects of the nondimensional constant $\left(a\right)$ on the skin friction constant in both x−direction and y−direction are highlighted in Table 2. It is detected that with an increase in the nondimensional constant $\left(a\right)$, there is no effect of $\left(a\right)$ on $Re_{x}^{\frac{1}{2}}C_{fx}$ and $Re_{y}^{\frac{1}{2}}C_{fy}$ such that $Re_{x}^{\frac{1}{2}}C_{fx},$ and $Re_{y}^{\frac{1}{2}}C_{fy}$ remains constant. In Table 3, our present work with Elbashbeshy et al. [29] and Sandeep et al. [30] is found to be in good agreement.

## 6. Conclusions

In the current article, a numerical investigation of three-dimensional hybrid nanomaterial micropolar fluid flow across an exponentially stretched sheet is conducted. By utilizing some appropriate transformations, the system of PDEs is transfigured into the design of ODEs and then solved via the bvp4c technique. The influences of different parameters are demonstrated through tables and graphs. However, some conclusions can be drawn from the current study.

- The velocity function is enhanced due to higher values of the solid nanoparticle concentration.
- The velocity function is enhanced due larger values of the micropolar parameter.
- The micropolar function $R\left(\eta \right)$ increases for higher values of the micropolar parameter and nanoparticle concentration.
- The micropolar function $R\left(\eta \right)$ declines for higher values of the micropolar parameter and nanoparticle concentration.
- The temperature function is enhanced for higher values of the solid nanoparticle concentration.
- Temperature function declines for higher values of the micropolar parameter.
- A comparison of the present work with those of Elbashbeshy et al. [29] and Sandeep et al. [30] when the rest of the physical parameters to be considered are zero are shown in Table 3.

## Figures and Tables

**Table 1:** Physical properties of hybrid nanofluid.

Viscosity	$\;\mu_{hnf}=\frac{\mu_{f}}{{\left(1-\phi_{1}\right)}^{2.5}{\left(1-\phi_{2}\right)}^{2.5}}$
Density	$\rho_{hnf}=\left[\left\{\left(1-\phi_{2}\right)\left(1-\phi_{1}\right)+\frac{\rho_{s_{1}}}{\rho_{f}}\phi_{1}\right\}+\frac{\rho_{s_{2}}}{\rho_{f}}\phi_{2}\right]$
Heat capacity	${\left(\rho c_{p}\right)}_{hnf}=\left[\left\{\left(1-\phi_{2}\right)\left(1-\phi_{1}\right)+\frac{{\left(\rho c_{p}\right)}_{s_{1}}}{{\left(\rho c_{p}\right)}_{f}}\phi_{1}\right\}+\frac{{\left(\rho c_{p}\right)}_{s_{2}}}{{\left(\rho c_{p}\right)}_{f}}\phi_{2}\right]$
Thermal conductivity	$\begin{array}{c} \frac{k_{hnf}}{k_{bf}}=\frac{k_{s_{2}}+k_{bf}\left(n-1\right)-\phi_{2}\left(k_{bf}-k_{s_{2}}\right)\left(n-1\right)}{k_{s_{2}}+\left(n-1\right)k_{bf}+\phi_{2}\left(k_{bf}-k_{s_{2}}\right)}\\ \text{And, }\frac{k_{bf}}{k_{f}}=\frac{k_{s_{1}\left(n-1\right)+k_{f}}-\phi_{1}\left(n-1\right)\left(k_{f}-k_{s_{1}}\right)}{\left(n-1\right)k_{f}+k_{s_{1}}+\phi_{1}\left(k_{f}-k_{s_{1}}\right)} \end{array}$

**Table 2:** Numerical values of $Re_{x}^{\frac{1}{2}}C_{fx}$ and $Re_{y}^{\frac{1}{2}}C_{fy}$ for $(Al_{2}O_{3}-Cu$ )/Water.

$\phi_{2}$	a	K	c	$Re_{x}^{\frac{1}{2}}C_{fx}$	$Re_{y}^{\frac{1}{2}}C_{fy}$
0.01	0.5	0.5	0.5	−2.4259	−1.5957
0.02				−2.6676	−1.7458
0.03				−2.9206	−1.9017
0.04				−3.1853	−2.0635
0.01	0.1			−2.4259	−1.5957
	0.3			−2.4259	−1.5957
	0.5			−2.4259	−1.5957
	0.7			−2.4259	−1.5957
	0.5	0.1		−2.2351	−1.1983
		0.3		−2.3285	−1.3996
		0.5		−2.4259	−1.5957
		0.7		−2.5248	−1.7870
		0.5	0.1	−2.2392	−0.2946
			0.3	−2.3340	−0.9212
			0.5	−2.4259	−1.5957
			0.7	−2.5150	−2.3161

**Table 3:** Comparison of the present work with Elbashbeshy et al. [29] and Sandeep et al. [30] when the rest of the physical parameters are zero.

Pr	Elbashbeshy et al. [29]	Sandeep et al. [30]	Present Work
0.72	0.7672800	0.76727610	0.76726891
1	0.9547800	0.95478230	0.95487123
2	1.4714600	1.47145810	1.4713654
3	1.8690700	1.86907210	1.8690612
5	2.5001300	2.50013010	2.5000987
10	3.6603700	3.66037230	3.66029876

## Data Availability

The data used to support the findings of this study are included within the article.
